# The College Chart for 1896

**Published:** 1896-03

**Authors:** 


### EDITORIAL.

### THE COLLEGE CHART FOR 1896.

THIS year will not close without witnessing severe and radical changes on the Veterinary College Chart of North America. The closing of some doors that should never have been opened seems now to be more than assured. The withdrawal of other schools from strong competition for place will almost prove a necessity. A discriminating, discerning, and educated public will draw severer lines than ever before. The better geographical distribution of these institutions will follow, and a higher, broader range of scientific instruction be demanded in places where the position of the schools seemed almost impregnable. The spirit of unrest stalks abroad in the land, and the appeal of the offspring to their college parents can no longer go unheeded. While it slumbered with but an occasional cloud of smoke, swiftly blown from sight, it gave little evidence of the depth of fire underneath, which has already broken forth in its fury at more than one point, and ere many more months have passed will he burning at others and only the protection of a higher, broader curriculum will quench the disastrous results.

The Eastern or New England States will retain the well-founded veterinary department at Harvard, and its value as an educational centre will grow stronger in attractions and worth year by year.

The Middle States, with no less than six schools to sustain and nourish, must change and concentrate her teaching power and centralize her valued and well-equipped instructors in fewer schools. New York State can hardly keep open doors at three points; all are no longer needed, and the profession will be stronger and of more worth to our people when centralization of these forces shall have been accomplished. There is doubtful place for two schools at Washington, save one becomes a post-graduate school.

Between the Allegheny Mountains and the Mississippi River no less than four schools are bidding for support, and this same territory has long been the great recruiting groun d for one of the Canadian schools. With but one exception, the course of instruction is too short in all these schools and the field of their employment to-day needs the highest grade of veterinarian that any of our schools can send forth. The sun of the narrow though well-equipped equine physician or surgeon has set, and from this time forth the needs of the hour will be veterinarians broadly and thorougly equipped in all the branches of sanitary police, milk and meat-inspection, breeding, control work, etc., and those schools not equipped to furnish such an education will be forced to close their doors for want of support. The field of the veterinarian is well filled to-day with a class of graduates who are only a slight step in advance of the self-made men so numerous in every State of our land.

With two schools beyond the Mississippi and one on the Pacific slope, it will be many years before there is need for any more in this section. These schools are all giving a graded course of three years, of six or eight months each, and are fully alive to the needs of a higher education for the future veterinarian.

The rush to establish new schools is over for a time; the yearning to pose before the public as a professor somewhat appeased; the belief that large monetary returns awaited but for the plucking has already left some sadder but wiser. The change has been swift, but not too soon; the losses will be best for the true growth of the profession and the survival of those who are well equipped for the great responsibilities of teachers, will find places of worth in strengthening the schools that shall remain after the storm that has so rudely awakened us all to the greater requirements demanded for the future successful veterinarian.

